# Systematic analyses of a novel circRNA-related miRNAs prognostic signature for Cervical Cancer

**DOI:** 10.1590/1678-4685-GMB-2021-0405

**Published:** 2022-06-24

**Authors:** Shasha Wang, Songying Zhang

**Affiliations:** 1 Zhejiang University, School of Medicine, Sir Run Run Shaw Hospital, Department of Obstetrics and Gynecology, Assisted Reproduction Unit, Hangzhou, China.

**Keywords:** Circular RNAs, competing endogenous RNAs, miRNAs, least absolute shrinkage and selection operator, prognostic signature

## Abstract

Accumulating evidences shed light on the important roles of Circular RNAs (circRNAs) acting as competing endogenous RNAs (ceRNAs) in cervical cancer (CC) biology. The present study aimed to identify a novel circRNA-related prognostic signature for CC. The expression data and clinical information of CC were downloaded from the Gene Expression Omnibus (GEO) datasets to identify the differential circRNAs expression. Based on the targeted miRNA prediction, circRNA-related miRNAs were detected in training group and validation group of The Cancer Genome Atlas (TCGA) dataset to construct the novel prognostic signature of CC with least absolute shrinkage and selection operator (LASSO). Moreover, the Kaplan-Meier (K-M) analysis was applied to test the model. In the present study, three differentially expressed circRNAs (hsa_circ_0001498, hsa_circ_0066147, and hsa_circ_0006948) were identified in GSE102686 and GSE107472. Then, with the criteria 25 predicted miRNAs were analyzed in TCGA datasets to calculate the prognostic signature. Furthermore, we developed a six-miRNA signature (hsa-miR-217, hsa-miR-30b-3p, hsa-miR-136-5p, hsa-miR-185-3p, hsa-miR-501-5p and hsa-miR-658) based on their expression level and coefficients. We performed a Pearson correlation analysis to screen 47 mRNAs which are negatively regulated by these six miRNAs. Functional enrichment analysis indicated these mRNAs were mainly enriched in cancer-related biology, such as regulation of transcription, signal transduction, and cell cycle. The present study provides novel insight for better understanding of circRNA-related ceRNA network in CC and facilitates the identification of potential biomarkers for prognosis.

## Introduction

Among females, cervical cancer (CC) takes the fourth place for both commonly diagnosed cancer (570,000 cases) and the leading cause of cancer death (311,000 deaths) across 20 world regions in 2018 (Bray *et al.,* 2018). There is a 10-fold variation in the incidence of CC by regions, which largely reflects human papillomavirus (HPV), human immunosuppression, parity, smoking, and oral contraceptive use (IARC Working Group on the Evaluation of Carcinogenic Risks to Humans, 2007; Tsikouras *et al.,* 2016; Olusola *et al.,* 2019). In spite of the development of early diagnosis and new targeted treatments, the five years overall survival (OS) rate of advanced-stage CC cases remains less than 40% (Wu *et al.,* 2019). Therefore, it is a great importance to identify the novel biomarkers to predict CC cases with poor prognosis.

Recent studies reveal that noncoding RNAs (ncRNAs) play important biological roles in carcinogenesis and development of cancers such as cell proliferation, differentiation and apoptosis (Romano *et al.,* 2017; Anastasiadou *et al.,* 2018; Chan and Tay, 2018). Circular RNAs (circRNAs), reported as by-products of spliceosome-mediated splicing errors by previous published studies (Shen *et al.,* 2015), and have been shown to exert novel crucial functions in the pathological progressions of various diseases, such as cardiovascular disease (Altesha *et al.,* 2019), nervous system disease (Ma *et al.,* 2020), digestive system disease (Sun *et al.,*, 2019). Because of the tissue-specific manner, circRNAs has drawn intense interest in their role in human diseases (Han B *et al.,* 2018). Increasing number of studies suggest that aberrant expressions of circRNAs have appeared to participate in the development and progression of various kinds of cancers, including CC (Zhang *et al.,* 2018; Verduci *et al.,* 2019; Li *et al.,* 2020). For instance, high circRNA_101996 expression level predicted poor outcomes and promoting tumor growth and metastasis by regulating miR-8075-TPX2 axis in CC (Song *et al.,* 2019). CircRNA_0000285 was overexpressed in CC samples and CC cells and identified as an oncogene by suppressing expression level of FUS in CC (Chen *et al.,* 2019).

The Competing Endogenous RNA (ceRNA) hypothesis was raised as a critical regulation mechanism between ncRNA and coding RNA (Salmena *et al.,* 2011). Recent studies have shown circRNAs can interact with the microRNA (miRNA) as miRNA sponges by miRNA-binding sites (MREs) (Hansen *et al.,* 2013), endogenously compete with mRNAs, and regulate protein translation (Abdelmohsen *et al.,* 2017). Moreover, the miRNAs sponge is one of the most important and widely investigated functions in circRNAs. MiRNA is another type of ncRNA that plays a key role by acting as cancer suppressors or oncogenes in various diseases (Mishra *et al.,* 2016). For example, by competing miR-326 to up-regulate the expression of ETS transcription factor ELK1 (ELK1), circ_0000515 enhanced proliferation and invasion but supressed apoptosis and autophagy of CC cells (Tang *et al.,* 2019). CircCLK3 was shown to promote the growth and metastasis of CC in vitro and in vivo by regulating miR-320a/FoxM1 axis (Hong *et al.,* 2019). Although various underlying function and mechanisms of circRNAs in CC have been characterized, the majority circRNAs contributing to biological properties of CC remain inconclusive. 

The aim of the present study was to identify novel circRNA-related-ceRNA signatures for CC prognosis through data mining of the public database such asGene Expression Omnibus (GEO, https://www.ncbi.nlm.nih.gov/geo/) and The Cancer Genome Atlas (TCGA, http://cancergenome.nih.gov). By conducting a comprehensive reanalysis of circRNA expression profile, we constructed circRNA-related ceRNA, and identified a novel circRNA-related-miRNA signature as a new candidate indicator which has the potential to predict the over survival in CC cases. We present the following article in accordance with the REMARK reporting checklist.

## Material and Methods

### Identification of RNA in GEO and TCGA dataset

CircRNA High-throughput sequencing and microarray data from 10 pairs CC tissues and paired-paracancerous cervical tissues were downloaded from the two public microarray CC GEO datasets (GSE102686 and GSE107472). The basic information for these two microarray circRNA profiles is summarized in Table 1. Normalized CC miRNA and mRNA raw data (level 3) and clinical data from 307 CC tissues were obtained from the TCGA Data portal. The exclusion criteria were set as follows: i) histologic diagnosis is not CC; ii) suffering of other malignancy except CC; iii) patients samples without complete data for analysis; and iv) overall survival (OS) >5 years. Overall, a total of 283 CC patients were included in this study. These data were reanalysed by using R software. 

**Table 1 -:** Basic information of the two microarray datasets from Gene Expression Omnibus**.**

Profile	RNA type	Platform	Organism	Experiment type	Sample size (T/N)	Region	Year
GSE102686	CircRNA	GPL19978	Homo sapiens	Non-coding RNA profiling by array	5/5	China	2017
GSE107472	CircRNA	GPL16791	Homo sapiens	Non-coding RNA profiling by high throughput sequencing	5/5	USA	2017

*Identification of differentially expressed circRNAs and target-regulation miRNA prediction*

In this study, the differential expression of circRNAs were determined by fold-change and associated P-values in GSE102686 and GSE107472, respectively. Fold change represents the difference in expression of each circRNA between CC tissues and paired-paracancerous cervical tissues. The criteria of the differentially expressed circRNAs were fold changes>1.5 or <0.67 and P<0.05. Then, the intersection of differentially expressed circRNAs was selected from GSE102686 and GSE107472. In addition, both miRanda (http://www.microrna.org/micror-na/home.do) and PITA (http://genie.weizmann.ac.il/pubs/mir07/mir07_data.html) were used to predict the circRNAs-interact miRNAs. 

### Construction of the CC-specific circRNA-related miRNAs prognostic signature

All CC cases in TCGA datasets were evenly randomized into training group and validation group. Based on the above circRNAs-interact miRNAs, CC-specific circRNA-related miRNAs were selected in TCGA database. The expression of miRNAs should assurance more than 60% of the amount of expression greater than zero. 

In the training group, Cox regression analysis with least absolute shrinkage and selection operator (LASSO) were performed to construct risk score to indicate the relative CC progression hazard for each case (Mao *et al.,* 2019). LASSO minimizes the sum of squares of residuals whose absolute values are less than constant. LASSO regularization contains a parameter λ to limit the number of selected features, where the larger λ retains more features. Lambda.min is used to determine the λ value, and Lambda determines the minimum average cross validation error (Birnbaum *et al.,* 2017). Afterward, a miRNA-related signature was determined by the sum value of miRNA expression profiles. According to the cutoff of risk score, cases were divided into High-risk group and Low-risk group. The hazard ratio (HR) and 95% confidence interval (CI) were assessed. 

### Predictive efficiency evaluation of the risk signature

Kaplan-Meier (K-M) plotter along with log-rank methods was further applied to compare the survival distributions between high‐risk and low‐risk groups (Sui *et al.,* 2018). The time‐dependent receiver operating characteristic (ROC) curves was plotted to assess the predictive accuracy of the risk score for time-dependent outcomes analysis using the R package “survival-ROC” (Heagerty *et al.,* 2000). Then univariate Cox regression analysis was applied to identify risk score-related miRNAs linked to OS.

### Risk score-related miRNAs functional assessment

The target genes of risk score-related miRNAs were predicted by mirTarBase (http://mirtarbase.cuhk.edu.cn/php/index.php), miRanda, Targetscan (http://www.targetscan.-org/mamm_31/), and miRWalk (http://mirwalk.umm.uniheidelberg.de/). To identify the target genes with prognostic characteristics, the univariate Cox regression analysis was also used to explore the relationship between the mRNAs and the OS of CC cases in TCGA datasets. Furthermore, we performed a Pearson correlation analysis to screen the negatively regulated mRNAs. To explore the possible pathways and biological processes of negatively regulated target genes, KEGG (Kyoto Encyclopedia of Genes and Genomes) pathways and GO (Gene Ontology) biological processes were analyzed through DAVID (the Database for Annotation, Visualization, and Integrated Discovery, http://david.abcc.ncifcrf.gov/) platform (Sui *et al.,* 2016). 

### The circRNAs and risk score-related miRNAs verification

This part was approved by the Ethics Committee of the Sir Run Run Shaw Hospital (20211018093606877). CC and normal control samples were obtained from biobank of Sir Run Run Shaw Hospital ZheJiang University School of Medicine. All patients had signed informed consent for donating their samples to biobank of Sir Run Run Shaw Hospital ZheJiang University School of Medicine. Total RNA was extraction from nine pairs CC tissues and paired-paracancerous cervical tissues with TRIzol reagent (Invitrogen, Carlsbad, CA, USA) according to the manufacture's protocol. U6 was used as an internal normalized reference to confirm reliability and validity of miRNAs. Reverse transcription reactions using A214 reverse transcription system kit (Genstar, Beijing, China) was conducted in two steps according to the manufacturer's protocol. Real-time PCR was performed to detect the expression level of the candidate miRNAs with the StepOne Plus™PCR System (Applied biosystems, Foster City, CA, USA). qRT-PCR was then performed with 2×RealStar Green Fast Mixture with ROX (Genstar, A303, Beijing, China) according to the manufacturer's protocol. The primer sequences were as follows: miR-185-3p, forward, 5′‐GATCACACTCTTGTGGTAGTTGC‐3′, reverse 5′‐CTCTTCCTTGCTCGTTGTTGGTAT‐3′, miR‐501-5p, forward, 5′‐TGCGCAATCCTTTGTCCCTGGG‐3′, reverse, 5′‐CCAGTGCAGGGTCCGAGGTATT‐3′, and U6, forward 5′‐CTCGCTTCGGCAGCACA‐3′, reverse 5′‐AACGCTTCACGAATTTGCGT‐3′. The PCR reaction components were 0.4 µL of cDNA, 10 µl of 2×RealStar Green Fast Mixture, 0.8 µl (1 µl/pmol) PCR primers, and 8.8 µL RNase-free water. The reaction was performed at 95˚C for 2 min, followed by 40 cycles of 95˚C for 15 s, 60˚C for 30 s and 72˚C for 30 s. A dissociation curve was analyzed from 60 to 95˚C. The Ct-value for each sample was calculated with the ∆∆Ct method, and the results of fold change were calculated by 2^-∆∆Ct^, where ∆∆Ct = (Ct_miRNAs_ - Ct_U6_ )_tumor_ - (Ct_miRNAs_ - Ct_U6_ )_paracancerous cervical tissues_. The study was conducted in accordance with the Declaration of Helsinki (as revised in 2013).

### Statistical analysis

Statistical analyses were performed using IBM SPSS 21.0 software (Chicago, USA). The results were expressed as mean±standard deviation (SD). Statistical significance was determined using the Student's t test or One-way Analysis of Variance (ANOVA) at the probability of P<0.05.

## Results

### Identification of differentially expressed circRNAs

**Figure 1-:**
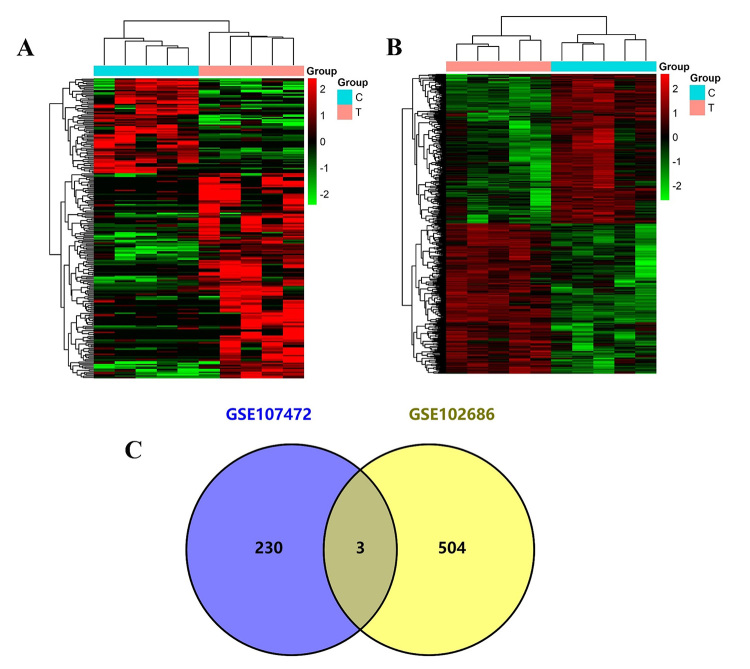
Differentially expressed circRNAs in GEO. (A) Differentially expressed circRNAs from GSE107472. (B) Differentially expressed circRNAs from GSE102686. (C) Intersection of differentially expressed circRNAs.

With the criteria of fold changes>1.5 or <0.67 and P<0.05, differentially expressed circRNAs profile consisting of 507 circRNAs, with 253 downregulated and 254 upregulated circRNAs, was identified from GSE102686 (Figure 1A). In GSE107472, 73 downregulated and 160 upregulated circRNAs were identified (Figure 1B). To further consolidate the data reliability, we selected three differentially expressed circRNAs (hsa_circ_0001498 downregulated, and hsa_circ_0066147 and hsa_circ_0006948 upregulated) from the intersection of the above two groups (Figure 1C).

*circRNAs target-regulation miRNA prediction*

Based on the above three circRNAs (hsa_circ_0001498, hsa_circ_0066147 and hsa_circ_0006948), a total of 244 and 534 miRNAs were predicted by miRanda and PITA, respectively. After merging these two results, 146 miRNAs were screened (Table S1). Furthermore, with the criteria of more than 60% of the amount of expression greater than zero, only 25 miRNAs left in TCGA datasets.

### Identification of a miRNA‐related prognostic signature

**Figure 2-:**
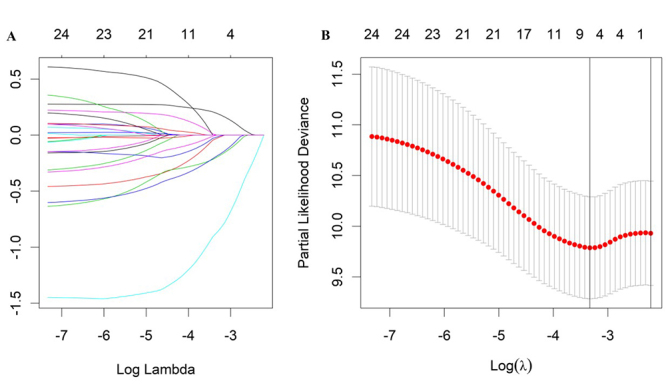
Coeﬃcients of selected features are shown by lambda parameter (A) LASSO coefficient profiles of the miRNAs associated with the overall survival of cervical cancer. (B) Plots of the cross-validation error rates.

283 CC cases from TCGA datasets were randomly divided into training group (n=142) and validation group (n=141). To identify the novel prognostic signature for CC cases, LASSO Cox regression model was implemented to analyze the data of miRNAs expression profiles in the training group. A batch of six miRNAs, hsa-miR-217, hsa-miR-30b-3p, hsa-miR-136-5p, hsa-miR-185-3p, hsa-miR-501-5p and hsa-miR-658, was selected as the most accurate prognostic predictor in the training group (Figure 2A). Further, we developed a six-miRNA signature based on the expression level and coefficients of these miRNAs. Risk score = 0.1944*hsa-miR-217+(-0.029*hsa-miR-30b-3p)+(-0.1975*hsa-miR-136-5p)+(-0.0179*hsa-miR-185-3p)+(-0.1869*hsa-miR-501-5p)+(-0.8713*hsa-miR-658) (Figure 2B). 

**Figure 3-:**
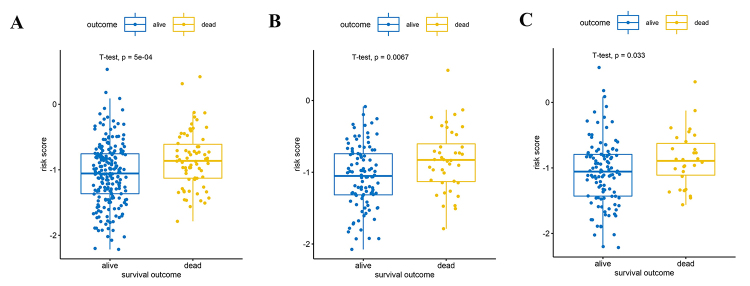
circRNA risk score analysis of 283 cervical cancer patients in TCGA cohort.(A) Risk score of circRNA signature in total cervical cancer cases. The value of risk score in the outcome of alive cases was significantly lower than dead cases (P<0.05). (B) Risk score of circRNA signature in training group. The value of risk score in the outcome of alive cases was significantly lower than dead cases (P<0.05). (C) Risk score of circRNA signature in validation group. The value of risk score in the outcome of alive cases was significantly lower than dead cases (P<0.05).283 cervical cases were randomly divided into training group (n=142) and validation group (n=141).

**Figure 4-:**
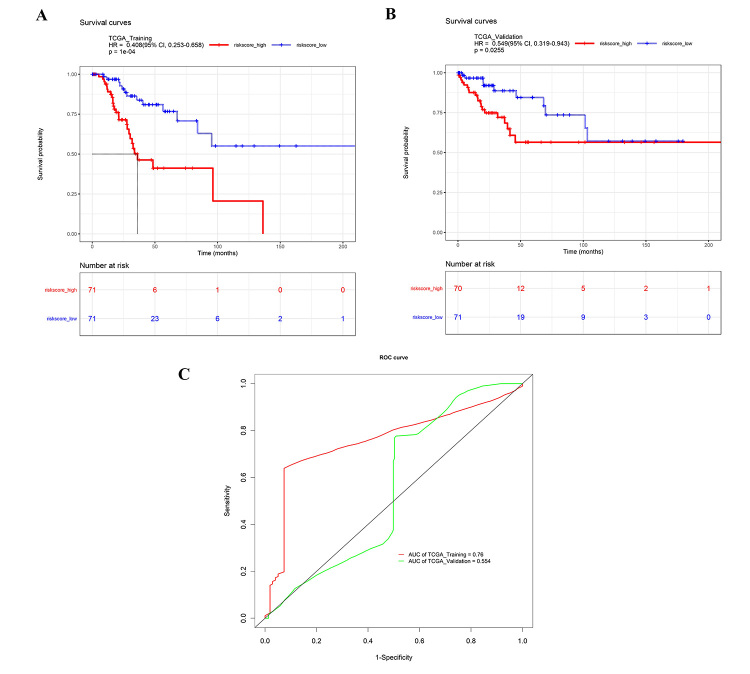
The six-miRNA prognostic signature of cervical cancer for outcome in cervical cancer patients in TCGA cohort. (A) The Kaplan-Meier curves for cervical cancer risk groups obtained from the TCGA cohort (training group, n=142) divided by the median cutoff point. Patients with high scores had poor outcome in terms of OS (P<0.05). (B) The Kaplan-Meier curves for cervical cancer risk groups obtained from the TCGA cohort (validation group, n=141) divided by the median cutoff point. Patients with high scores had poor outcome in terms of OS (P<0.05).(C) The risk score shown by the time-dependent ROC curve for predicting 5-year survival in training group and validation group. 283 cervical cases were randomly divided into training group (n=142) and validation group (n=141).

**Figure 5 -:**
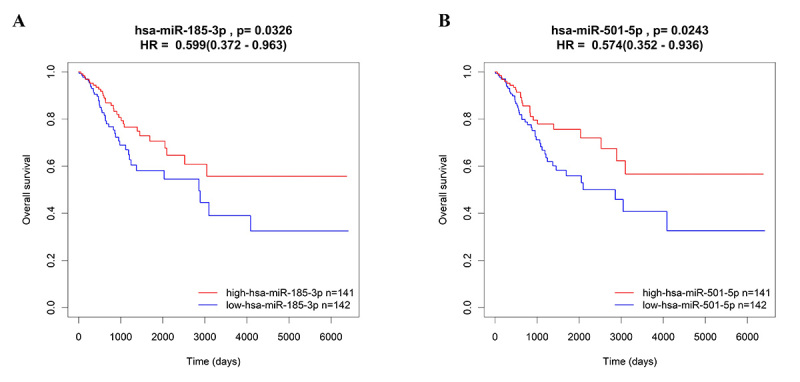
The Kaplan-Meier curves for miRNA obtained from 283 cervical cancer patients in TCGA cohort. (A) The Kaplan-Meier curves for miR-185-3p obtained from the TCGA cervical cancer cohort (n=283) divided by the median cutoff point. Patients with high expression of miR-185-3p had better outcome in terms of OS (P<0.05). (B) The Kaplan-Meier curves for miR-501-5p obtained from the TCGA cervical cancer cohort (n=283) divided by the median cutoff point. Patients with high expression of miR-501-5p had better outcome in terms of OS (P<0.05).

The risk score for each case in all CC cases, training group and validation group was calculated and plotted. As shown in Figure 3, the value of risk score in the outcome of alive cases was significantly lower than dead cases in all CC cases, training group and validation group (P<0.05). The robustness of the miRNA signature was detected by evaluating their ability to classify low-risk group and high-risk group in training group and validation group. Firstly, the miRNA signature-based risk score for each CC case was calculated. Then, CC cases were divided into low-risk group or high-risk group according to the median value. K-M curves were plotted along with log rank p-test, to compare the OS of the two groups. According to the results, significant differences in K-M survival analysis were similarly observed in low-risk group and high-risk group separated by the miRNA signature in training group (Figure 4A) and validation group (Figure 4B). The time-dependent ROC curves were used to determine the sensitivity and specificity of the predictive model. As shown in Figure 4C, predict accuracy of the novel miRNA signature in training group and validation group. In addition, univariate Cox proportional hazards regression showed that two (hsa-miR-185-3p and hsa-miR-501-5p) of these above six miRNAs in the TCGA datasets were identified with a significant prognostic value (Figure 5).

### Prediction of miRNA targets

**Figure 6-:**
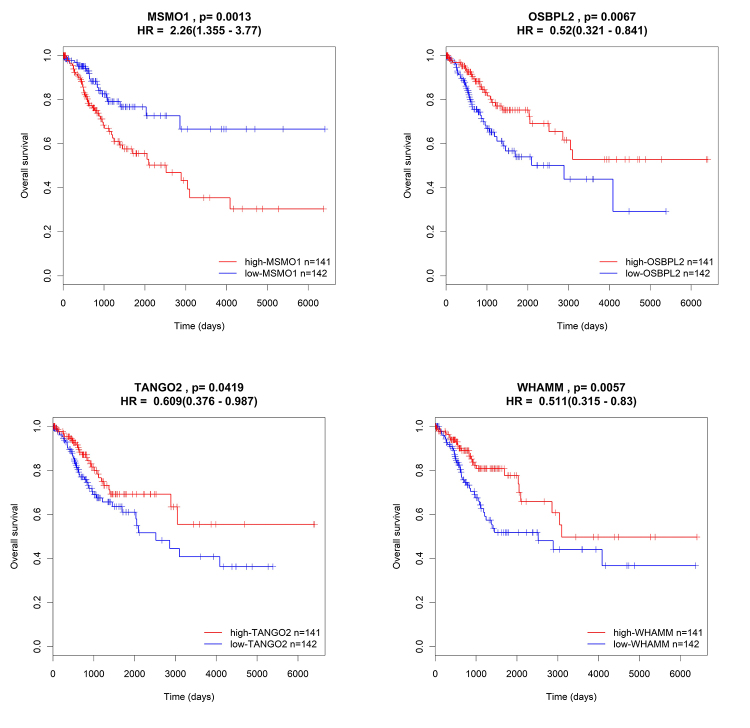
The Kaplan-Meier test of negatively regulated mRNAs (MSMO1, OSBPL2, TANGO2 and WHAMM) for the OS.

To inquire into the KEGG pathways and GO biological processes relevant to the six-prognostic signature-related miRNAs, we first predicted the targeted correlation between miRNA and mRNAs with mirTarBase, miRanda, Targetscan, and miRWalk. 1226, 12690, 7083, and 8356 targeted mRNAs were identified respectively (Figure S1). The results of univariate Cox regression between these above mRNAs and the OS of CC cases in TCGA datasets were shown in Figure S2. Then, based on negative regulation theory (Fabian *et al.,* 2010), we performed a Pearson correlation analysis to screen 47 negatively regulated mRNAs (Table S2). MSMO1, OSBPL2, TANGO2, and WHAMM in these 47 mRNAs were significantly associated with OS (P<0.05) (Figure 6).

### Functional enrichment analysis for miRNAs signature

**Figure 7 -:**
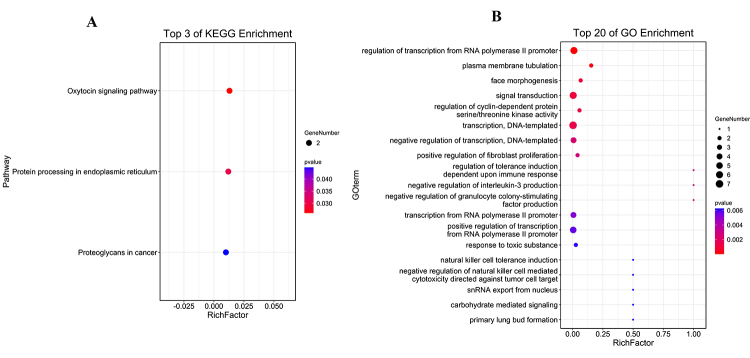
GO and KEGG term analysis of potential genes related to negatively regulated mRNAs. (A)Three top enrichment of KEGG pathways. (B) Twenty top enrichment of GO terms. The Rich factor shows the degree of enrichment, which was calculated by the formula: (the number of selected genes in a term/total number of selected genes)/(the total number of genes in a term of the database/ the total number of genes in the database). The node size represents the number of selected genes, and color represents the P-value of the enrichment analysis.

Furthermore, these above 47 negatively regulated mRNAs were utilized for KEGG pathways and GO biological processes enrichment analysis by DAVID. Pathway enrichment analysis indicated three significantly enriched pathways were Oxytocin signaling pathway, Protein processing in endoplasmic reticulum, and Proteoglycans in cancer (Figure 7A). For GO analysis, when considering CC, the mRNAs were enriched in regulation of transcription, signal transduction, regulation of immune response, and cell cycle (Figure 7B).

### The expression of miRNA verification.

**Figure 8-:**
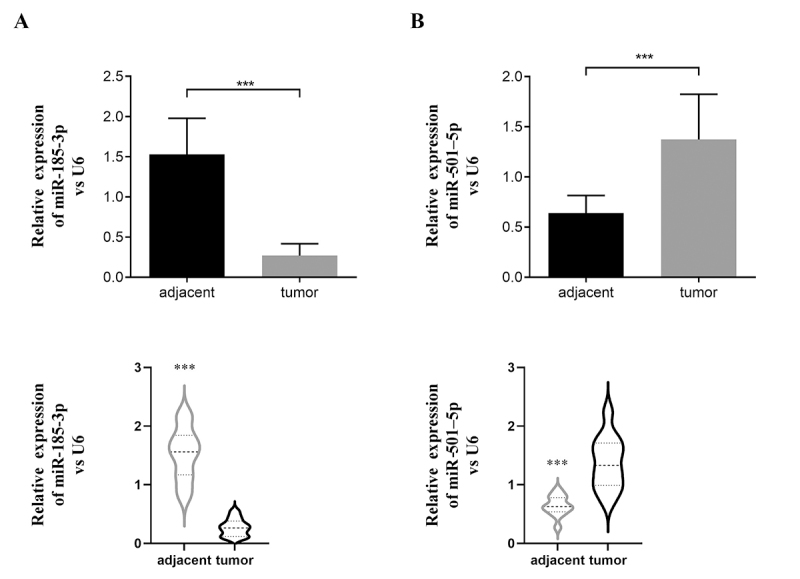
Results of qRT-PCR validation of miR-185-3p (A) and miR-501-5p (B) in cervical cancer tissues and paired-paracancerous tissues (upper panel: histogram, lower panel: violin plot).

Finally, hsa-miR-185-3p was downregulated expressed in CC tissues, while hsa-miR-501-5p was upregulated expressed in CC tissues detected by qRT-PCR (9 pairs of CC and adjacent tissues were tested) (Figure 8).

## Discussion

In the present study, the circRNAs expression profiles of CC cases downloaded from GEO datasets were reanalyzed and three CC-specific circRNAs were identified. According to the targeted-miRNAs prediction of these three circRNAs, CC cases in TCGA datasets were randomly separated to training group and validation group to discover a prognostic six‐miRNA signature related to OS by LASSO Cox regression model. LASSO is considered to be a novel high‐dimensional variable selection method for regression (Tibshirani, 1997). LASSO is extremely important to help select parsimonious variables in the application of increasing the efficiency of CC prognosis prediction. Furthermore, LASSO can also improve the prediction accuracy and enhance numerical stability and model interpretability. Risk score was calculated with the regression coefficients that generated by the Cox regression analysis. Based on the prognostic signature, the risk score was calculated for each CC case and the training group was divided into low‐risk group and high‐risk group. Then prognostic value of the six‐miRNA signature was verified in the validation group. K-M analysis showed that distinct separation between the survival curves of the high‐risk group and low‐risk group that classified by the same criteria in both training group and validation group was observed. These results indicated this miRNA signature in CC cases is high reproducible.

To further assess the predictive efficiency based on risk score, time‐dependent ROC curves was introduced into our study. The results showed that the novel prognostic signature has a better predictive accuracy. Moreover, the risk score based on these six-miRNAs could be used as a new indicator for the prognosis of CC cases. Generally, miRNAs act as the ceRNAs which regulate gene expression through mRNA processing and degradation (Wang *et al.,* 2017). Their related negatively regulated mRNAs were predicted in various software and TCGA datasets to analyze the possible biological processes. The functional enrichment analysis suggested that these above mRNAs were enriched in cancer‐related signaling pathways such as proteoglycans in cancer and protein processing in endoplasmic reticulum, and biological process such as signal transduction, regulation of immune response, and cell cycle. The results of functional enrichment clarified the possible effects of the CC-specific circRNAs related miRNAs in the prognostic signature.

It has been demonstrated that non-coding RNAs play an important role in the progression of cervical cancer. For instance, for 158 cervical specimens, including 38 normal, 52 cervical intraepithelial neoplasia and 68 CC tissues, the result showed a remarkable increase of miR-25, miR-92a, and miR-378 with lesion progression but no obvious change of miR-22, miR-29a, and miR-100 among the HPV-infected tissues (Wang *et al.,* 2014). It was also reported that miR-18a targets the tumor suppressor STK4/MST1, which is necessary for HPV-positive cervical cancer transformation (Morgan *et al.,* 2020). Long noncoding RNAs (lncRNAs) are also involved in the regulation of cellular processes that are dysregulated during carcinogenesis (Anastasiadou *et al.,* 2018). For example, the long non-coding RNA DINO is acutely expressed in human papillomavirus-positive cervical cancer cells and reactivates the dormant TP53 tumor suppressor through ATM/CHK2 signaling (Sharma and Munger, 2020a). In addition, the expression of long non-coding RNA DINO is mediated by KDM6A, which leads to the stability of TP53 tumor suppressor in cells expressing human papillomavirus 16 E7 (Sharma and Munger, 2020b).

Previous studies have demonstrated that miR-185-3p is downregulated in CC tissues and regulated the proliferation and self-renewal ability of CC stem cells by repressing FOXP3 expression in CC cells (Zhang *et al.,* 2021). Besides, unexpressed miR‐185 promoted apoptosis, suppressed cell migration and invasion, and related with better OS (Gao *et al.,* 2020). Upregulated miR-501-5p was detected in gastric cancer tissues and cell lines with poor OS in gastric cancer patients by targeted and suppressed multiple repressors of the WNT/β-catenin signaling (Fan *et al.,* 2016). A few studies have reported the role of miR-217 in CC. Zhu *et al.* (2019) found miR-217 was downregulated in CC tissues and CC cells. Upregulated miR-217 suppressed the viability, migration and invasion in CC cells (Dong *et al.,* 2018; Yin and Ren, 2019; Zhu *et al.,* 2019). MiR-30b-3p has been reported to be a potential targeted regulatory of GPNMB to regulate the prognosis of gastric cancer patients (Yao *et al.,* 2020). MiR-136 has been shown to be downregulated in CC tissues (Han M-S *et al.,* 2018), and regulated by lncRNA FOXP4-AS1 to involve the CC progression (Zhao *et al.,* 2020). Xu *et al.* (2020) reported circNFIC suppresses breast cancer progression by sponging miR-658. It has been demonstrated that circRNAs act as competitive endogenous RNAs (ceRNAs) and play an important role in the progression of cervical cancer. CircRNA8924 is highly expressed in CC tissue and can be considered a competitive endogenous RNA of the miR-518d-5p/519-5p family to promote the malignant biological behavior of CC cells (Liu *et al.,* 2018). circ_0067934 and circ_0005576 could promote CC progression via miR-545/EIF3C axis and circ_0005576/miR-153-3p/KIF20A axis, respectively (Hu *et al.,* 2019; Ma *et al.,* 2019). However, there is no report on the prognosis of these six miRNAs with CC. Thus, the six-miRNAs prognostic signature might be a novel prognostic biomarker in CC treatment and survival evaluation.

Although the results of the present study might have remarkable prognostic implications, some limitations should also be considered. First, a longer follow-up time should be verified in this study. Second, in addition to the data in the GEO and TCGA datasets, other experimental methods should apply to validate the results in the present study. Third, the function of the three circRNAs and six miRNAs should be elucidated in further studies.

In conclusion, the present study identified a CC-specific circRNA-related six-miRNA signature could be used as a potential outcome predictor for CC cases via re-analyzing the genome-wide RNA expression profile datasets from GEO and TCGA database with a ceRNA network. Future functional studies are required to elucidate the roles of these circRNAs and miRNAs in CC. 

## Supplementary material

The following online material are available for this article:

Table S1 - The intersected miRNAs predicted by targeted circRNAs.

Table S2 - miRNAs targeting CC-specific mRNAs.

Figure S1 - Predicted intersection of targeted mRNA with mirTarBase, miRanda, Targetscan, and miRWalk.

Figure S2 - The Kaplan-Meier test of six-miRNA targeted mRNAs.
