# Supplementary material for: Systematic analyses of a novel circRNA-related miRNAs prognostic signature for Cervical Cancer
Source: Genet Mol Biol. 2022 Jun 24;45(2):e20210405. doi: 10.1590/1678-4685-GMB-2021-0405 (PMC9241030; doi:10.1590/1678-4685-GMB-2021-0405)
Supplement: Table S1 - [file 1415-4757-GMB-45-2-e20210405-s1.pdf]

Genetics and Molecular Biology
DOI: https://doi.org/10.1590/1678-4685-GMB-2021-0405

Supplementary Material to: "Systematic analyses of a novel circRNA-related miRNAs prognostic signature for Cervical Cancer"

Table S1 - The intersected miRNAs predicted by targeted circRNAs

circRNA	circRNA_style	Predicted miRNA
hsa_circ_0066147	up	hsa-let-7b-5p
hsa_circ_0066147	up	hsa-let-7c-5p
hsa_circ_0066147	up	hsa-let-7d-5p
hsa_circ_0066147	up	hsa-miR-10b-3p
hsa_circ_0001498	down	hsa-miR-122-5p
hsa_circ_0066147	up	hsa-miR-1229-5p
hsa_circ_0001498	down	hsa-miR-1231
hsa_circ_0066147	up	hsa-miR-1249-5p
hsa_circ_0006948	up	hsa-miR-1254
hsa_circ_0066147	up	hsa-miR-1254
hsa_circ_0006948	up	hsa-miR-1264
hsa_circ_0001498	down	hsa-miR-1266-3p
hsa_circ_0066147	up	hsa-miR-1273g-3p
hsa_circ_0006948	up	hsa-miR-1296-3p
hsa_circ_0066147	up	hsa-miR-1307-3p
hsa_circ_0001498	down	hsa-miR-1321
hsa_circ_0066147	up	hsa-miR-1343-5p
hsa_circ_0006948	up	hsa-miR-136-5p
hsa_circ_0001498	down	hsa-miR-139-5p
hsa_circ_0066147	up	hsa-miR-15a-3p
hsa_circ_0066147	up	hsa-miR-185-3p
hsa_circ_0066147	up	hsa-miR-196b-5p
hsa_circ_0066147	up	hsa-miR-217
hsa_circ_0001498	down	hsa-miR-2467-3p
hsa_circ_0066147	up	hsa-miR-29a-3p
hsa_circ_0066147	up	hsa-miR-3065-3p
hsa_circ_0006948	up	hsa-miR-30b-3p
hsa_circ_0006948	up	hsa-miR-3116
hsa_circ_0066147	up	hsa-miR-3116
hsa_circ_0066147	up	hsa-miR-3150a-3p
hsa_circ_0006948	up	hsa-miR-3151-5p
hsa_circ_0066147	up	hsa-miR-3155a
hsa_circ_0066147	up	hsa-miR-3169
hsa_circ_0001498	down	hsa-miR-3187-5p
hsa_circ_0006948	up	hsa-miR-3197
hsa_circ_0001498	down	hsa-miR-323b-5p
hsa_circ_0066147	up	hsa-miR-326
hsa_circ_0066147	up	hsa-miR-328-5p
hsa_circ_0006948	up	hsa-miR-3612
hsa_circ_0066147	up	hsa-miR-3612
hsa_circ_0001498	down	hsa-miR-3650
hsa_circ_0066147	up	hsa-miR-3663-3p
hsa_circ_0006948	up	hsa-miR-3689a-3p
hsa_circ_0006948	up	hsa-miR-3689a-5p

circRNA	circRNA_style	Predicted miRNA
hsa_circ_0006948	up	hsa-miR-3689b-3p
hsa_circ_0006948	up	hsa-miR-3689b-5p
hsa_circ_0006948	up	hsa-miR-3689c
hsa_circ_0006948	up	hsa-miR-3689e
hsa_circ_0006948	up	hsa-miR-3689f
hsa_circ_0001498	down	hsa-miR-3692-3p
hsa_circ_0006948	up	hsa-miR-370-3p
hsa_circ_0006948	up	hsa-miR-3908
hsa_circ_0066147	up	hsa-miR-3929
hsa_circ_0001498	down	hsa-miR-410-5p
hsa_circ_0066147	up	hsa-miR-4254
hsa_circ_0001498	down	hsa-miR-4277
hsa_circ_0006948	up	hsa-miR-432-5p
hsa_circ_0006948	up	hsa-miR-4443
hsa_circ_0066147	up	hsa-miR-4457
hsa_circ_0006948	up	hsa-miR-4471
hsa_circ_0006948	up	hsa-miR-4531
hsa_circ_0006948	up	hsa-miR-4654
hsa_circ_0066147	up	hsa-miR-4656
hsa_circ_0001498	down	hsa-miR-4658
hsa_circ_0006948	up	hsa-miR-4663
hsa_circ_0066147	up	hsa-miR-4667-5p
hsa_circ_0006948	up	hsa-miR-4681
hsa_circ_0066147	up	hsa-miR-4685-5p
hsa_circ_0006948	up	hsa-miR-4686
hsa_circ_0066147	up	hsa-miR-4690-3p
hsa_circ_0006948	up	hsa-miR-4717-3p
hsa_circ_0066147	up	hsa-miR-4725-3p
hsa_circ_0006948	up	hsa-miR-4731-5p
hsa_circ_0001498	down	hsa-miR-4741
hsa_circ_0066147	up	hsa-miR-4741
hsa_circ_0006948	up	hsa-miR-4758-5p
hsa_circ_0001498	down	hsa-miR-4779
hsa_circ_0001498	down	hsa-miR-4801
hsa_circ_0001498	down	hsa-miR-485-5p
hsa_circ_0066147	up	hsa-miR-486-3p
hsa_circ_0006948	up	hsa-miR-490-3p
hsa_circ_0066147	up	hsa-miR-5006-5p
hsa_circ_0006948	up	hsa-miR-501-5p
hsa_circ_0001498	down	hsa-miR-5093
hsa_circ_0066147	up	hsa-miR-513a-5p
hsa_circ_0001498	down	hsa-miR-5189-5p
hsa_circ_0006948	up	hsa-miR-5194
hsa_circ_0006948	up	hsa-miR-5196-3p
hsa_circ_0066147	up	hsa-miR-5196-5p
hsa_circ_0006948	up	hsa-miR-520a-5p
hsa_circ_0066147	up	hsa-miR-520g-3p
hsa_circ_0066147	up	hsa-miR-520h
hsa_circ_0006948	up	hsa-miR-525-5p
hsa_circ_0066147	up	hsa-miR-541-5p
hsa_circ_0006948	up	hsa-miR-558
hsa_circ_0001498	down	hsa-miR-574-5p
hsa_circ_0006948	up	hsa-miR-584-3p
hsa_circ_0066147	up	hsa-miR-597-3p
hsa_circ_0066147	up	hsa-miR-6081

circRNA	circRNA_style	Predicted miRNA
hsa_circ_0066147	up	hsa-miR-638
hsa_circ_0006948	up	hsa-miR-650
hsa_circ_0066147	up	hsa-miR-6515-5p
hsa_circ_0066147	up	hsa-miR-658
hsa_circ_0066147	up	hsa-miR-668-5p
hsa_circ_0066147	up	hsa-miR-671-5p
hsa_circ_0006948	up	hsa-miR-6728-3p
hsa_circ_0006948	up	hsa-miR-6736-5p
hsa_circ_0066147	up	hsa-miR-6737-5p
hsa_circ_0066147	up	hsa-miR-6754-5p
hsa_circ_0066147	up	hsa-miR-6763-5p
hsa_circ_0006948	up	hsa-miR-6765-3p
hsa_circ_0006948	up	hsa-miR-6778-5p
hsa_circ_0006948	up	hsa-miR-6779-5p
hsa_circ_0006948	up	hsa-miR-6780a-3p
hsa_circ_0006948	up	hsa-miR-6780a-5p
hsa_circ_0066147	up	hsa-miR-6780b-5p
hsa_circ_0006948	up	hsa-miR-6785-5p
hsa_circ_0001498	down	hsa-miR-6790-5p
hsa_circ_0001498	down	hsa-miR-6797-5p
hsa_circ_0066147	up	hsa-miR-6797-5p
hsa_circ_0006948	up	hsa-miR-6800-3p
hsa_circ_0066147	up	hsa-miR-6813-5p
hsa_circ_0006948	up	hsa-miR-6821-3p
hsa_circ_0066147	up	hsa-miR-6821-5p
hsa_circ_0066147	up	hsa-miR-6828-5p
hsa_circ_0001498	down	hsa-miR-6830-5p
hsa_circ_0006948	up	hsa-miR-6839-5p
hsa_circ_0066147	up	hsa-miR-6840-3p
hsa_circ_0066147	up	hsa-miR-6846-5p
hsa_circ_0066147	up	hsa-miR-6847-5p
hsa_circ_0066147	up	hsa-miR-6848-5p
hsa_circ_0006948	up	hsa-miR-6852-3p
hsa_circ_0006948	up	hsa-miR-6865-5p
hsa_circ_0001498	down	hsa-miR-6866-5p
hsa_circ_0006948	up	hsa-miR-6871-5p
hsa_circ_0001498	down	hsa-miR-6876-3p
hsa_circ_0006948	up	hsa-miR-6882-3p
hsa_circ_0001498	down	hsa-miR-6884-5p
hsa_circ_0066147	up	hsa-miR-6885-5p
hsa_circ_0001498	down	hsa-miR-6891-5p
hsa_circ_0006948	up	hsa-miR-6893-3p
hsa_circ_0006948	up	hsa-miR-7106-5p
hsa_circ_0006948	up	hsa-miR-7109-5p
hsa_circ_0006948	up	hsa-miR-766-5p
hsa_circ_0066147	up	hsa-miR-7706
hsa_circ_0001498	down	hsa-miR-7846-3p
hsa_circ_0066147	up	hsa-miR-8075
hsa_circ_0066147	up	hsa-miR-891a-3p
hsa_circ_0001498	down	hsa-miR-922
hsa_circ_0006948	up	hsa-miR-93-3p
hsa_circ_0066147	up	hsa-miR-939-5p
