# Supplementary material for: Systematic analyses of a novel circRNA-related miRNAs prognostic signature for Cervical Cancer
Source: Genet Mol Biol. 2022 Jun 24;45(2):e20210405. doi: 10.1590/1678-4685-GMB-2021-0405 (PMC9241030; doi:10.1590/1678-4685-GMB-2021-0405)
Supplement: Table S2 - [file 1415-4757-GMB-45-2-e20210405-s2.pdf]

Genetics and Molecular Biology
DOI: https://doi.org/10.1590/1678-4685-GMB-2021-0405

Supplementary Material to: "Systematic analyses of a novel circRNA-related miRNAs prognostic signature for Cervical Cancer"

Table S2 - miRNAs targeting CC-specific mRNAs

miRNA	mRNA	Pearson R	P-Value
hsa-miR-217	FOSL2	-0.1331	0.0252
hsa-miR-217	RAP2B	-0.1218	0.0405
hsa-miR-30b-3p	ADAMTS4	-0.1258	0.0344
hsa-miR-30b-3p	AKIP1	-0.1299	0.0288
hsa-miR-30b-3p	CCL22	-0.1552	0.0089
hsa-miR-30b-3p	CDCP1	-0.1425	0.0165
hsa-miR-30b-3p	CDKN1A	-0.1308	0.0279
hsa-miR-30b-3p	CLEC7A	-0.166	0.0051
hsa-miR-30b-3p	CRISPLD2	-0.1815	0.0022
hsa-miR-30b-3p	HAVCR2	-0.1813	0.0022
hsa-miR-30b-3p	RDH10	-0.1205	0.0428
hsa-miR-30b-3p	SLC35F6	-0.1232	0.0383
hsa-miR-30b-3p	TANGO2	-0.1211	0.0418
hsa-miR-30b-3p	TLCD2	-0.1196	0.0444
hsa-miR-136-5p	CDCP1	-0.1205	0.0428
hsa-miR-136-5p	NRIP3	-0.1219	0.0404
hsa-miR-136-5p	NUDT19	-0.1251	0.0354
hsa-miR-136-5p	SCNM1	-0.165	0.0054
hsa-miR-136-5p	SERTAD1	-0.117	0.0493
hsa-miR-185-3p	FBXW2	-0.1711	0.0039
hsa-miR-185-3p	FOXK1	-0.1426	0.0164
hsa-miR-185-3p	GOLGA3	-0.1358	0.0223
hsa-miR-185-3p	ILF3	-0.1435	0.0157
hsa-miR-185-3p	OSBPL2	-0.1421	0.0167
hsa-miR-185-3p	PHAX	-0.1369	0.0213
hsa-miR-185-3p	PLEKHA1	-0.1506	0.0112
hsa-miR-185-3p	PPP1R12B	-0.1244	0.0365
hsa-miR-185-3p	RAB21	-0.117	0.0493
hsa-miR-185-3p	RPH3AL	-0.1308	0.0278
hsa-miR-185-3p	TLCD2	-0.1357	0.0224
hsa-miR-185-3p	TRIM13	-0.1178	0.0477
hsa-miR-185-3p	TRIM66	-0.1884	0.0015
hsa-miR-185-3p	WHAMM	-0.1532	0.0098
hsa-miR-185-3p	ZBTB3	-0.1696	0.0042
hsa-miR-185-3p	ZNF250	-0.1708	0.004
hsa-miR-185-3p	ZNF770	-0.1269	0.0328
hsa-miR-501-5p	ATF6	-0.1177	0.0479
hsa-miR-501-5p	CDCP1	-0.1254	0.035
hsa-miR-501-5p	CDKN1A	-0.1277	0.0318
hsa-miR-501-5p	EHD2	-0.1574	0.008
hsa-miR-501-5p	FOSL2	-0.1312	0.0273
hsa-miR-501-5p	MSMO1	-0.1209	0.0421
hsa-miR-501-5p	PPP1R12B	-0.1211	0.0418
hsa-miR-501-5p	RAP2B	-0.1222	0.04

miRNA	mRNA	Pearson R	P-Value
hsa-miR-501-5p	TNS4	-0.1166	0.05
hsa-miR-658	AHR	-0.1265	0.0333
hsa-miR-658	FOXK1	-0.121	0.042
hsa-miR-658	GOLGA3	-0.1904	0.0013
hsa-miR-658	MAZ	-0.14	0.0185
hsa-miR-658	NFAT5	-0.1287	0.0304
hsa-miR-658	PPFIBP1	-0.127	0.0327
hsa-miR-658	RNF24	-0.1646	0.0055
hsa-miR-658	SLC35F6	-0.1179	0.0475
hsa-miR-658	SSR3	-0.1875	0.0015
hsa-miR-658	TMEM120B	-0.1544	0.0093
hsa-miR-658	ZCCHC24	-0.1441	0.0152
hsa-miR-658	ZNF587	-0.1289	0.0301
