# Supplementary material for: Systematic analyses of a novel circRNA-related miRNAs prognostic signature for Cervical Cancer
Source: Genet Mol Biol. 2022 Jun 24;45(2):e20210405. doi: 10.1590/1678-4685-GMB-2021-0405 (PMC9241030; doi:10.1590/1678-4685-GMB-2021-0405)
Supplement: Figure S2 - [file 1415-4757-GMB-45-2-e20210405-s4.pdf]

Genetics and Molecular Biology
DOI: https://doi.org/10.1590/1678-4685-GMB-2021-0405

Supplementary Material to: "Systematic analyses of a novel circRNA-related miRNAs prognostic signature for Cervical Cancer"

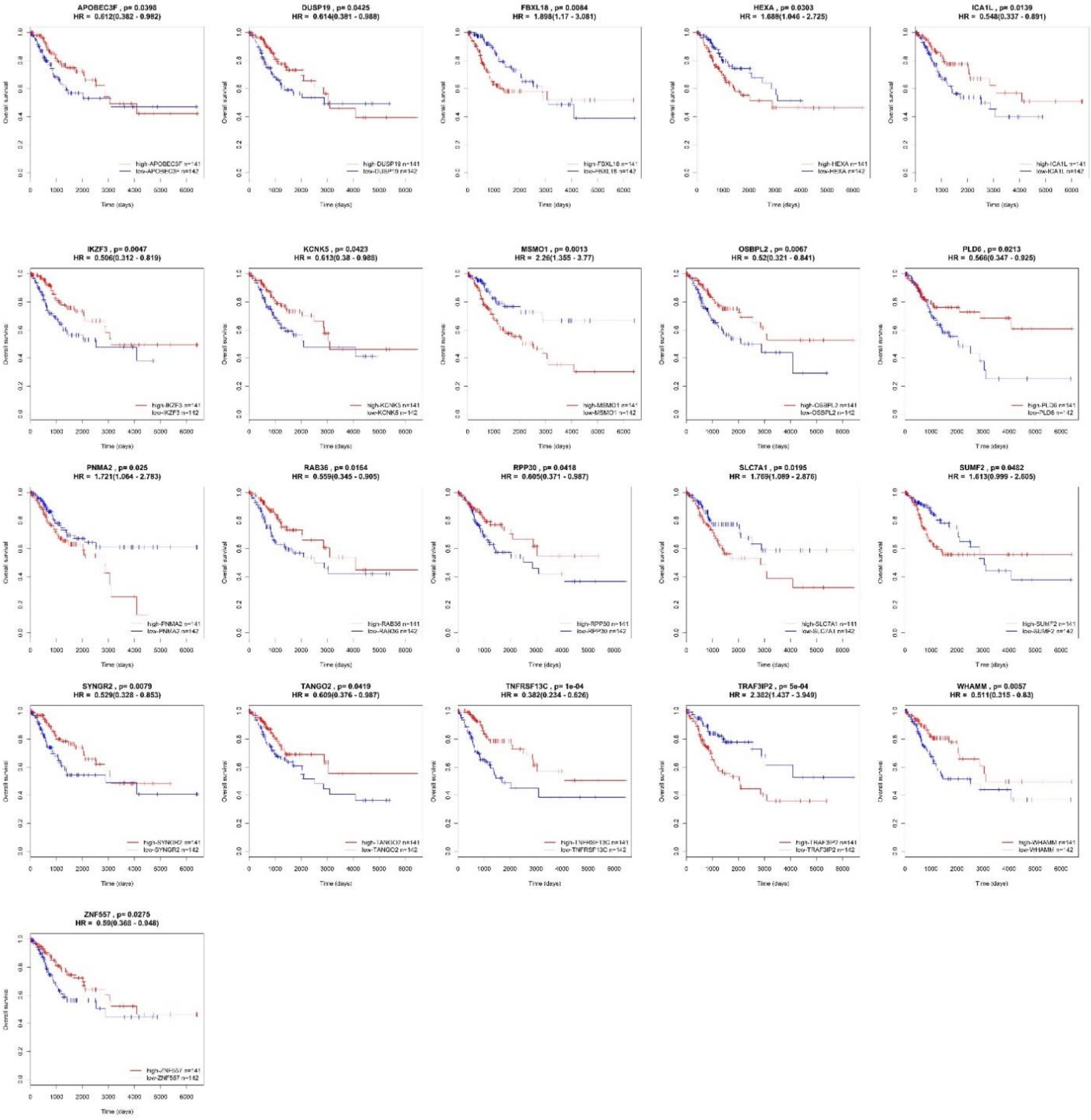

Figure S2 – The Kaplan-Meier test of six-miRNA targeted mRNAs
